# Quantitative pixel-wise measurement of myocardial blood flow: The impact of surface coil-related field inhomogeneity and a comparison of methods for its correction

**DOI:** 10.1186/s12968-015-0117-1

**Published:** 2015-02-11

**Authors:** Christopher A Miller, Li-Yueh Hsu, Allison Ta, Hannah Conn, Susanne Winkler, Andrew E Arai

**Affiliations:** Department of Health and Human Services, Advanced Cardiovascular Imaging Laboratory, National Heart, Lung and Blood Institute, National Institutes of Health, Bethesda, MD 20892-1061 USA

**Keywords:** Cardiovascular magnetic resonance, Perfusion, Myocardial blood flow, Quantification, Field inhomogeneity, Surface coil intensity correction

## Abstract

**Background:**

Surface coil-related field inhomogeneity potentially confounds pixel-wise quantitative analysis of perfusion CMR images. This study assessed the effect of surface coil-related field inhomogeneity on the spatial variation of pixel-wise myocardial blood flow (MBF), and assessed its impact on the ability of MBF quantification to differentiate ischaemic from remote coronary territories. Two surface coil intensity correction (SCIC) techniques were evaluated: 1) a proton density-based technique (PD-SCIC) and; 2) a saturation recovery steady-state free precession-based technique (SSFP-SCIC).

**Methods:**

26 subjects (18 with significant CAD and 8 healthy volunteers) underwent stress perfusion CMR using a motion-corrected, saturation recovery SSFP dual-sequence protocol. A proton density (PD)-weighted image was acquired at the beginning of the sequence. Surface coil-related field inhomogeneity was approximated using a third-order surface fit to the PD image or a pre-contrast saturation prepared SSFP image. The estimated intensity bias field was subsequently applied to the image series. Pixel-wise MBF was measured from mid-ventricular stress images using the two SCIC approaches and compared to measurements made without SCIC.

**Results:**

MBF heterogeneity in healthy volunteers was higher using SSFP-SCIC (24.8 ± 4.1%) compared to PD-SCIC (20.8 ± 3.0%; p = 0.009), however heterogeneity was significantly lower using either SCIC technique compared to analysis performed without SCIC (36.2 ± 6.3%). In CAD patients, the difference in MBF between remote and ischaemic territories was minimal when analysis was performed without SCIC (0.06 ± 0.91 mL/min/kg), and was substantially lower than with either PD-SCIC (0.50 ± 0.63 mL/min/kg; p = 0.013) or with SSFP-SCIC (0.63 ± 0.89 mL/min/kg; p = 0.005). In 6 patients, MBF quantified without SCIC was artifactually higher in the stenosed coronary territory compared to the remote territory. PD-SCIC and SSFP-SCIC had similar differences in MBF between remote and ischaemic territories (p = 0.145).

**Conclusions:**

This study demonstrates that surface coil-related field inhomogeneity can confound pixel-wise MBF quantification. Whilst a PD-based SCIC led to a more homogenous correction than a saturation recovery SSFP-based technique, this did not result in an appreciable difference in the differentiation of ischaemic from remote coronary territories and thus either method could be applied.

## Background

Quantitative analysis of perfusion cardiovascular magnetic resonance (CMR) appears to offer advantages over qualitative assessment, including more accurate evaluation of ischaemic burden, particularly in patients with multivessel disease, and higher reproducibility [1,2]. Pixel-wise quantitative analysis, in which myocardial blood flow (MBF) can be resolved at the level of approximately 30 μL of myocardium, provides a more physiological assessment of MBF than segmental analysis, allowing sub-segmental and transmural variations in MBF to be elucidated [3].

Recent advances in CMR pulse sequences and multi-element surface coils have improved the signal-to-noise ratio (SNR) for perfusion CMR. However, these improvements come with a trade-off in signal intensity homogeneity. Inhomogeneous sensitivity profiles of the phased array surface coils lead to spatial variation in signal intensity that can potentially confound quantitative perfusion measurements. In order to quantify pixel-wise MBF from perfusion CMR images, the arterial input function (AIF) is deconvolved from the myocardial dynamic contrast enhancement curves [4]. Since a single AIF is used for the entire myocardium, it is important to obtain uniform measurement of signal intensity over the entire myocardium. The impact of surface coil-related field inhomogeneity, and methods for its correction, on pixel-wise MBF quantification have not been assessed. More fundamentally, the impact of surface coil-related field inhomogeneity on the ability of quantitative perfusion analysis (segmental or pixel-wise) to detect coronary artery disease, has not been evaluated.

First, this study aimed to assess the effect of surface coil-related field inhomogeneity on spatial variation in MBF, and to evaluate the impact of two surface coil intensity correction (SCIC) techniques; a proton density-based technique and a saturation recovery steady-state free precession-based technique. The second, and main, aim of the study was to assess the impact of surface coil-related field inhomogeneity on the ability of pixel-wise MBF quantification to differentiate ischaemic from remote territories in patients with confirmed significant coronary artery disease, and to compare the effect of the two SCIC techniques.

## Methods

### Study population

Twenty-six subjects were recruited, including 18 patients with significant obstructive coronary artery disease (CAD) in 1 or 2 major epicardial coronary arteries and 8 healthy volunteers. Significant CAD was defined as ≥ 70% coronary luminal narrowing as demonstrated on invasive coronary angiography with qualitative analysis. Since the study aimed to evaluate the difference in measured MBF between ischaemic and remote coronary territories, patients with significant disease in all 3 major epicardial arteries, and thus no remote territory, were not included. Healthy volunteers had no known history of cardiovascular disease and a Framingham risk score of less than 1%. The study was approved by the National Heart, Lung and Blood Institute (NHLBI) Review Board, and all subjects gave written informed consent.

### CMR perfusion image acquisition

CMR was performed on a 1.5 Tesla scanner (Magnetom Espree, Siemens Healthcare, Erlangen, Germany), with a 12-element phased-array coil to evaluate myocardial perfusion during stress and at rest. Subjects were asked to abstain from caffeinated products for at least 24 hours prior to scanning. Patients with CAD underwent CMR within 40 days of angiography. Stress was achieved using Regadenoson, administered as a 400 mcg bolus over 10 seconds followed by a 10 mL saline flush. Stress perfusion imaging was performed 70 seconds after Regadenoson bolus. Aminophylline was administered after stress imaging was completed and rest perfusion imaging was performed twenty minutes later. Stress and rest imaging were each performed with a 0.05 mmol/kg bolus of gadolinium-based contrast agent (gadopentetate dimeglumine; Gd-DTPA; Magnevist; Berlex Laboratories, Wayne, NJ, USA) diluted to provide injections of equal volumes and flushed with saline at 5 mL/sec flow rate (Medrad, Indianola, PA, USA).

A saturation recovery-prepared steady-state free precession (SSFP) sequence [5] was used to acquire perfusion images at three slice locations (base, mid, and apex) every R-R interval for a period lasting 60 heartbeats during stress and at rest. Image motion correction was performed on all perfusion images after image reconstruction [6]. Typical imaging parameters included a saturation preparation pulse, readout excitation flip angle 50°, repetition time (TR) 2.3 ms, echo time (TE) 1.1 ms, bandwidth 1085 Hz/pixel, acquisition matrix 128 × 80, field of view (FOV) 360 × 270 mm, slice thickness 8 mm, and temporal resolution 92 ms with parallel imaging acceleration factor of 2. A proton density-weighted reference image was acquired at the beginning of perfusion imaging, using a small magnetization flip angle (5°) and no saturation preparation pulse, in order to facilitate surface coil intensity correction (see later section). A separate saturation prepared, low resolution image with FLASH readout, acquisition matrix 48 × 64, temporal resolution 60 ms, was acquired at the beginning of each RR interval at basal slice level for AIF assessment [5].

### Perfusion CMR analysis

All image analysis was performed using custom software developed in Interactive Data Language (Exelis Visual Information Solutions, Boulder, Colorado, USA). The process of quantitative pixel-wise perfusion image analysis has been described in detail previously [3]. In brief, endocardial and epicardial borders of the left ventricular (LV) myocardium were manually traced on the perfusion image series for myocardial region of interest (ROI) analysis. An additional ROI was drawn in the blood pool of the low resolution image series for extraction of the AIF. Next, a surface coil intensity correction was applied to the entire perfusion image series using one of the techniques described below. Rigid and non-rigid image registration was then performed to further reduce motion artifacts. Finally, pixel-wise myocardial time-signal intensity curves were extracted and quantified using a model constrained deconvolution in order to obtain pixel-wise MBF maps.

### Comparison of surface coil intensity correction techniques

**Figure 1:**
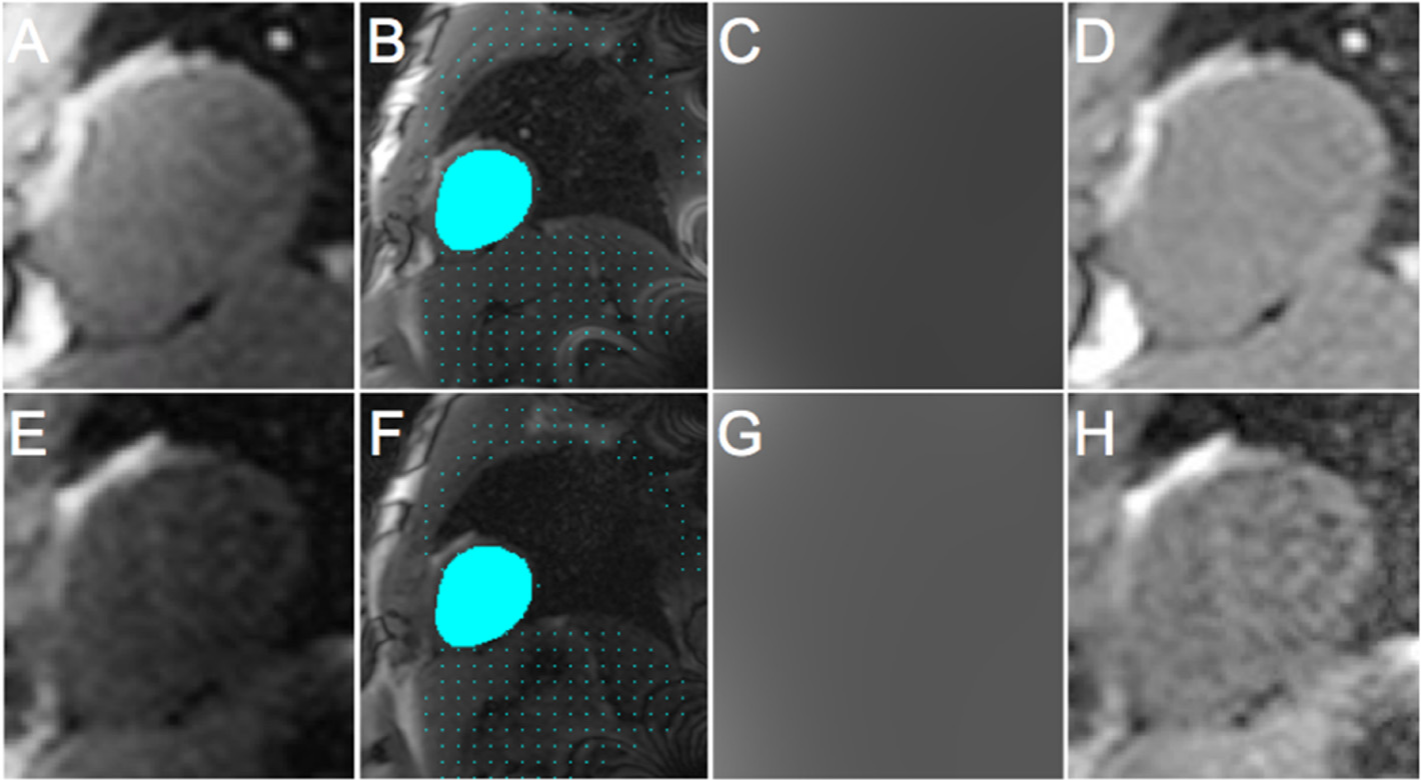
**Process of surface coil intensity correction (SCIC).** The original proton density (PD)-weighted image is displayed in **(A)** and the original first saturation prepared steady-state free precession (SSFP) image from the same subject is displayed in **(E)**, zoomed to focus on the heart. Myocardial (blue mask) and body (blue dots) regions of interest were manually drawn on the proton density image **(B)** or the first saturation prepared SSFP image **(F)**. The body region of interest was drawn such that it excluded regions with signal intensities that were markedly higher (e.g. fat) or lower (e.g. lungs) than myocardial signal intensity. Surface coil-related field inhomogeneity was then approximated using a third-order surface fit to myocardial and body signal intensities in the PD or SSFP images in order to generate an intensity bias field (**C** and **G** respectively). This was subsequently applied to the perfusion image series. The corrected PD and SSFP images are displayed in **D** and **H** respectively. The coefficient of variation of myocardial signal intensities (blue mask) for the corrected PD and SSFP images (i.e. **D** and **H** respectively) were compared (see Results).

In order to estimate surface coil-related field inhomogeneity, a myocardial ROI and a body ROI were manually drawn on the proton density image or on a pre-contrast baseline saturation prepared SSFP image (Figure 1). For the myocardial ROI, the epicardial cardiac border was traced. For the body ROI, the outer border of the body was traced excluding regions with signal intensities that were markedly higher (e.g. fat) or lower (e.g. lungs) than myocardial signal intensity. The myocardial and body ROIs provided segmentation of similar signal intensity tissue for estimating surface coil-related field inhomogeneity. Surface coil-related field inhomogeneity was then approximated using a third-order surface fit to myocardial and body signal intensities of the proton density image (PD-SCIC) or the baseline saturation prepared SSFP image (SSFP-SCIC) [7]. The estimated intensity bias field was subsequently applied to the perfusion image series for pixel-wise MBF quantification. Perfusion analysis was also performed without surface coil intensity correction (No-SCIC), in which only baseline normalization was performed. The following assessments were made on mid-ventricular stress images in order to compare the impact of the different SCIC techniques:

1. The homogeneity of myocardial signal intensity following surface coil intensity correction was evaluated by measuring the variation (coefficient of variation) in myocardial signal intensity on the corrected PD-weighted and pre-contrast baseline saturation prepared SSFP images in all subjects (Figure 1).
2. MBF heterogeneity was evaluated in healthy volunteers, who would be expected to have relatively homogenous MBF (see Discussion). Spatial heterogeneity, defined as the standard deviation of pixel-wise MBF divided by mean pixel MBF [8], was calculated. The difference between septal and lateral wall MBF was also assessed.
3. The ability of pixel-wise MBF quantification to differentiate ischaemic from remote territories was assessed by measuring the difference in MBF between ischaemic and remote territories in patients with confirmed significant CAD. For consistency, standard AHA/ACC coronary territories were used (anteroseptum and anterior wall = left anterior descending artery; anterolateral and inferolateral walls = circumflex artery; inferior wall and inferoseptum = right coronary artery [9]).

### Statistical analysis

Statistical analysis was performed using SPSS (IBM, USA; v20). Continuous variables are expressed as mean ± standard deviation unless stated. The coefficient of variation of myocardial signal intensity in surface coil intensity corrected PD and saturation prepared SSFP images was compared using a paired t-test, with p < 0.05 considered statistically significant. MBF spatial heterogeneity, the difference in MBF between septal and lateral walls and the difference in MBF between remote and ischaemic territories for each SCIC technique were compared using a paired t-test.

## Results

Perfusion imaging was performed successfully in 26 subjects. Subject characteristics are summarised in Table 1. For patients with CAD, the mean time interval between invasive coronary angiography and CMR perfusion imaging was 13 ± 15 days.

**Table 1:** **Subject characteristics**

	**CAD patients n = 18**	**Healthy volunteers n = 8**
Male	11 (61%)	7 (88%)
Age	58 ± 12	24 ± 10
Hypertension	12 (67%)	-
Hyperlipidemia	15 (83%)	-
Diabetes	4 (22%)	-
Smoker (ex-, current)	8 (44%), 2 (11%)	-
PCI	4 (22%)	-
CABG	1 (6%)	-
MI	3 (17%)	-
Extent of CAD		
1 vessel	10 (56%)	-
2 vessel	8 (44%)	-
Distribution of CAD		
LAD	14 (78%)	-
Cx	3 (17%)	-
RCA	9 (50%)	-

PCI indicates percutaneous coronary intervention; CABG coronary artery bypass surgery; MI previous history of myocardial infarction, CAD significant coronary artery disease; LAD left anterior descending coronary artery; Cx circumflex coronary artery; RCA right coronary artery.

### Signal intensity variation in SCIC corrected images

There was significantly greater variation in myocardial signal intensity in the saturation prepared SSFP surface coil intensity corrected images (coefficient of variation 20 ± 7%) compared to the PD corrected images (10 ± 5%; p < 0.001).

### MBF heterogeneity in healthy volunteers

**Figure 2:**
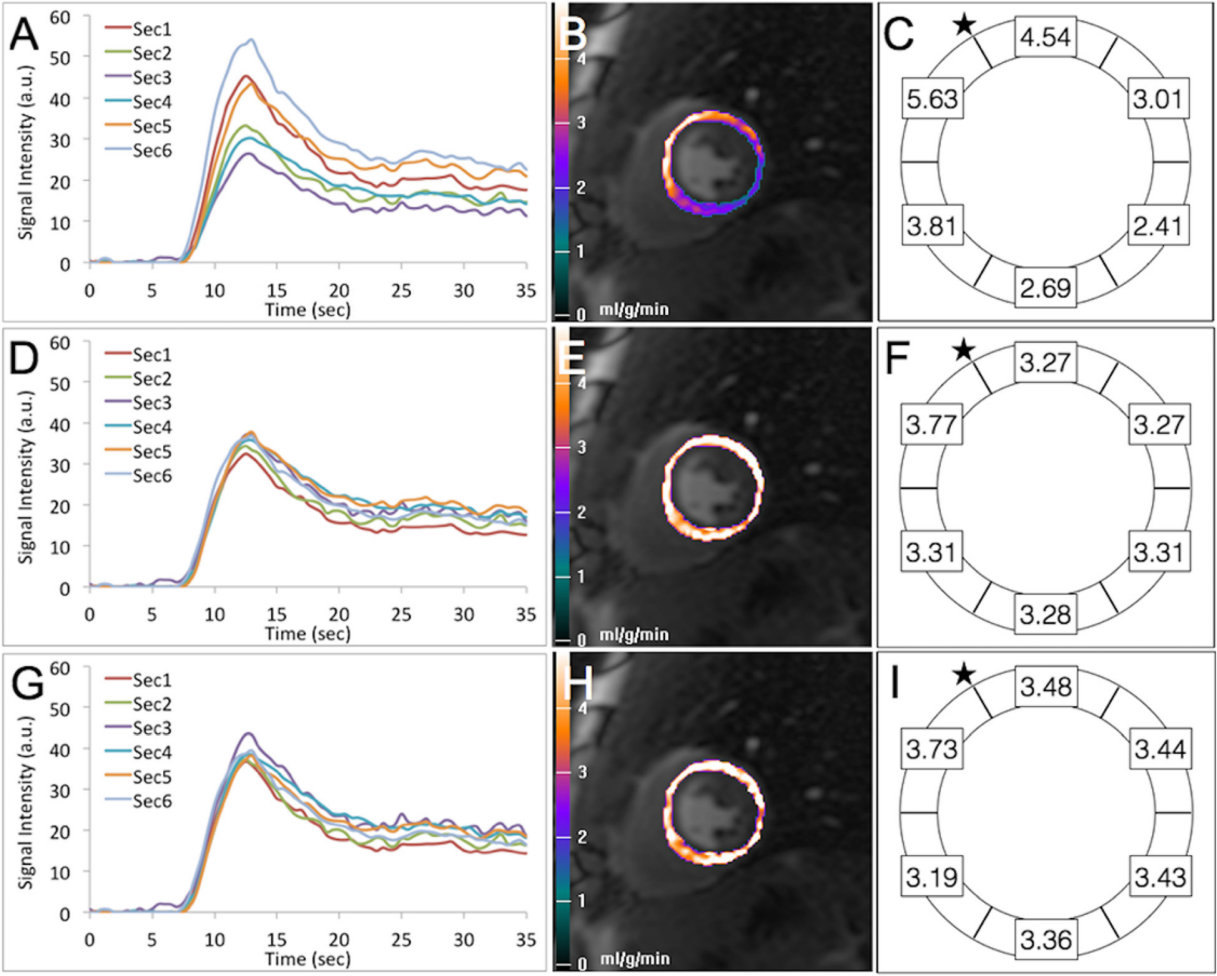
**Myocardial blood flow heterogeneity in a healthy volunteer. (A)** Segmental myocardial timesignal intensity curves with no surface coil intensity correction, and the corresponding myocardial blood flow (MBF) pixel map **(B)**. Considerable segmental variation is seen in the time-signal intensity curves which corresponds to marked heterogeneity in MBF, with MBF seen to be much higher in the anteroseptum compared to the inferolateral wall. **(C)** Mean measured segmental MBF (mL/min/kg) without surface coil intensity correction in healthy volunteers, demonstrating considerable regional variation, with MBF seen to be higher in the anterior and anteroseptal segments compared to the inferior and inferolateral segments (star represents anterior RV septal insertion point). In the same subject, after proton density **(D and E)** and saturation prepared SSFP **(G and H)** surface coil intensity correction, segmental time-signal intensity curves and MBF are much more homogenous. Mean segmental MBF is also considerably more homogenous after proton density **(F)** and saturation prepared SSFP **(I)** surface coil intensity correction. (Segmental rather than pixel-wise time-signal intensity curves are shown for ease of visual interpretation). Sec1 = anterior, Sec2 = anterolateral, Sec3=inferolateral, Sec4= inferior, Sec5 = inferoseptum, Sec6 = anteroseptum.

There was significantly lower MBF heterogeneity in healthy volunteers using PD-SCIC (20.8 ± 3.0%) compared to SSFP-SCIC (24.8 ± 4.1%; p = 0.009). Nevertheless, MBF heterogeneity was substantially lower using either PD-SCIC or SSFP-SCIC compared to when the analysis was performed without surface coil intensity correction (36.2 ± 6.3%; p < 0.001 compared with PD-SCIC; p = 0.001 compared with SSFP-SCIC; Figure 2). Indeed, when the analysis was performed without surface coil intensity correction, MBF was calculated to be 60% higher in the septum compared to the lateral wall (absolute difference of 1.70 ± 0.74 mL/min/kg). This difference was significantly greater than when PD-SCIC (-0.12 ± 0.51 mL/min/kg; p < 0.001) or SSFP-SCIC (-0.32 ± 0.43 mL/min/kg; p < 0.001) were used (Figure 2). The difference between septal and lateral wall MBF using PD-SCIC compared to SSFP-SCIC was of borderline significance only (p = 0.047).

### MBF in remote versus ischaemic coronary territories

**Figure 3:**
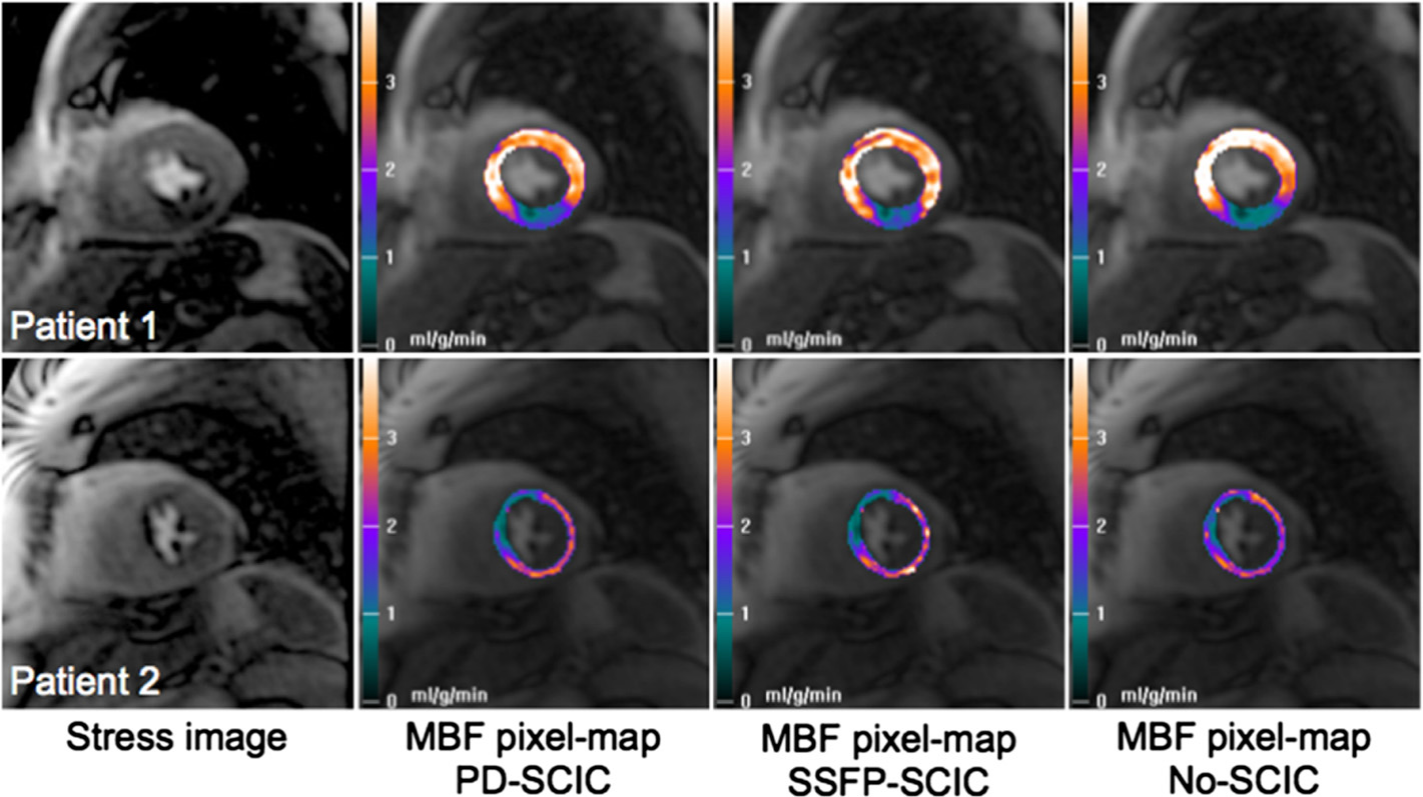
**Myocardial blood flow in patients with significant coronary artery disease.** Examples of concordance between different surface coil intensity correction techniques. **Patient 1;** 90% proximal right coronary artery (RCA) stenosis. A perfusion defect is evident in the inferior wall on the pixel-wise myocardial blood flow (MBF) maps generated using all surface coil intensity correction (SCIC) techniques. **Patient 2;** 90% proximal left anterior descending coronary artery (LAD) stenosis. A perfusion defect is evident in the anteroseptum and anterior wall on the pixel-wise MBF maps generated using all SCIC techniques.

**Figure 4:**
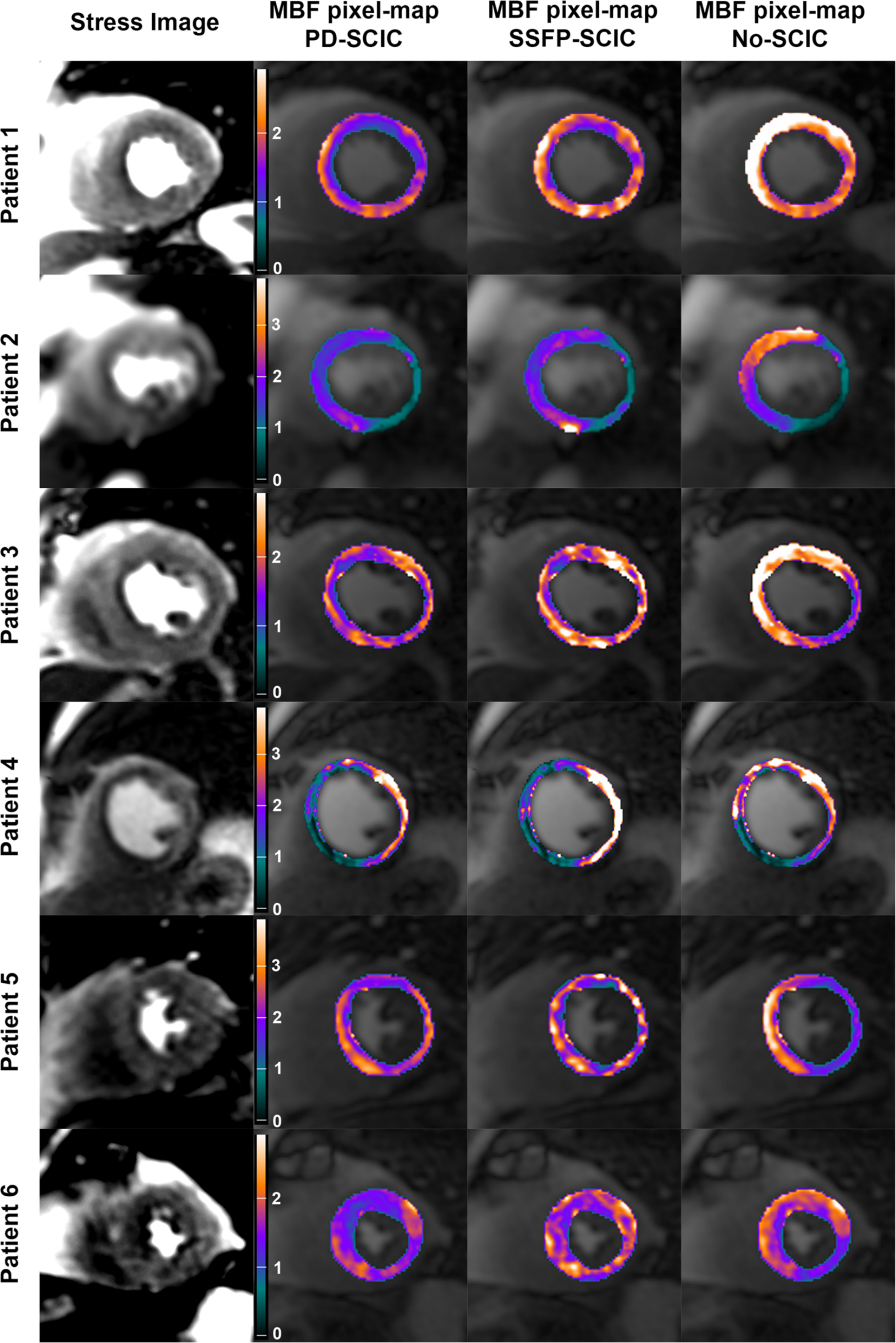
**Examples of concordances and discordances between coronary anatomy and myocardial blood flow (MBF) related to surface coil intensity correction (SCIC) method. Patient 1** has an 80% proximal LAD stenosis which is associated with reduced MBF in the anterior, anteroseptal, and anterolateral segments and well represented on the proton density weighted SCIC (PD-SCIC) pixel map. The perfusion defect is present but less obvious, on the saturation prepared steady-state free precession SCIC (SSFP-SCIC) pixel map. However on the pixel map quantified without SCIC (No-SCIC), MBF is erroneously highest in LAD territory and lower in the RCA territory. **Patient 2** has an occluded dominant circumflex obtuse marginal artery and a 90% proximal LAD stenosis. All pixel maps show severely decreased MBF in the circumflex distribution. However, the No-SCIC pixel map shows erroneously increased MBF in the anterior wall. The PDSCIC and SSFP-SCIC depict expected low MBF in the LAD and circumflex territories. **Patient 3** has a 75% ostial LAD stenosis. Low MBF is evident in the anteroseptum and anterior wall on the PD-SCIC and SSFP-SCIC pixel maps as expected. On the No-SCIC pixel map, MBF is erroneously highest in LAD territory and lower in the inferior and inferolateral walls. **Patient 4** has an 80% proximal LAD stenosis and 80% mid RCA stenosis. The PD-SCIC and SSFP-SCIC pixel maps depict low MBF in keeping with LAD and RCA disease. While the No-SCIC pixel map correctly detects low MBF in the RCA territory, it misleadingly shows essentially normal LAD MBF. **Patient 5** has a 75% proximal LAD stenosis. A subtle decrease in MBF is evident in the anteroseptum and anterior segment on the PD-SCIC pixel maps. This MBF abnormality is less obvious on the SSFPSCIC pixel map due to higher MBF heterogeneity. On the No-SCIC pixel map, MBF is erroneously highest in the anteroseptum and anterior wall, and incorrectly lowest in the inferior and lateral walls. **Patient 6** has a 90% proximal LAD stenosis. A perfusion defect is evident in the anteroseptum and anterior segment on the PD-SCIC pixel map but less obvious on the SSFP-SCIC pixel map due to heterogeneity of MBF. On the No-SCIC pixel map, MBF erroneously appears lowest in the inferior wall.

MBF in remote and ischaemic coronary territories in patients with CAD quantified using each SCIC technique is summarised in Table 2, and examples of MBF maps demonstrating concordance between SCIC techniques are shown in Figure 3. When the analysis was performed without surface coil intensity correction, the difference in MBF between remote and ischaemic territories was minimal (0.06 ± 0.91) and was substantially lower than with either PD-SCIC (0.50 ± 0.63 mL/min/kg; p = 0.013) or with SSFP-SCIC (0.63 ± 0.89 mL/min/kg; p = 0.005). In 6 patients, all with significant disease in the LAD artery, MBF quantified without SCIC was higher in the LAD territory than in the remote territory (i.e. the opposite of what was correct; Figure 4). There was no significant difference in body mass index (30.5 ± 9.3 vs. 29.9 ± 5.0 kg/m^2^; p = 0.88), body surface area (2.0 ± 0.27 vs. 1.90 ± 0.15 m^2;^ p = 0.46), weight (88.6 ± 26.3 vs. 82.9 ± 11.8 kg; p = 0.53) and height (171 ± 8.4 cm vs. 167 ± 9.1 cm; p = 0.40) between these 6 patients and the other 12 CAD patients. The difference in MBF between remote and ischaemic territories using PD-SCIC was not significantly different to that using SSFP-SCIC (p = 0.145).

**Table 2:** **Myocardial blood flow in remote and ischaemic coronary territories quant** **ified using each surface coil intensity correction technique in patients with coronary artery disease**

	**Remote territory MBF (mL/min/kg)**	**Ischaemic territory MBF (mL/min/kg)**	**Difference (mL/min/kg)**
PD-SCIC	2.24 ± 0.78	1.75 ± 0.53	0.50 ± 0.63
SSFP-SCIC	2.46 ± 1.03	1.84 ± 0.60	0.63 ± 0.89
No-SCIC	2.31 ± 1.05	2.25 ± 0.99	0.06 ± 0.91

See text for details. MBF indicates myocardial blood flow; PD-SCIC perfusion quantification performed using a proton density-based surface coil intensity correction technique; SSFP-SCIC perfusion quantification performed using a saturation recovery steady-state free precession-based surface coil intensity correction technique; No-SCIC perfusion quantification performed without surface coil intensity correction.

## Discussion

The main finding of this study is that surface coil-related field inhomogeneity can confound MBF quantification and lead to entirely inaccurate assessment of CAD. Performing a surface coil intensity correction is therefore an important part of CMR MBF quantification. In addition, this study demonstrated that surface coil intensity correction performed using a PD-weighted image led to a more homogenous correction than using a saturation recovery SSFP image, but the study showed that either surface coil intensity correction method could be useful.

Phased array surface coils provide higher signal to noise and the ability to perform parallel imaging, however these advantages come with the trade off of increased signal inhomogeneity relating to differences in coil sensitivity profiles. As demonstrated by Hoffman et al., who performed saturation recovery SSFP cardiac perfusion imaging with a 12 element coil in 13 subjects, higher signal intensities are generally observed in the anterior or ventral regions (anterior and anteroseptal segments) of the heart compared to the dorsal regions (inferior and inferolateral) [10]. In keeping with these findings, measured MBF appeared considerably higher in the septum compared to the lateral wall in healthy volunteers in the current study when perfusion analysis was performed without SCIC.

Surface coil intensity correction is particularly challenging for cardiac imaging because of the distance between the heart and surface coil, and the wide range of signal intensities, with abrupt changes, related to the anatomical structures close to the heart (chest wall, lung air, liver etc). Murakami et al. [11] described four techniques to correct for surface coil related field inhomogeneity. The first involves calculation of the coil’s sensitivity profile from the Biot-Savart law using knowledge of the coil’s size, shape, and position relative to the patient [12]. In the second, a homogeneous phantom is studied with the surface coil before imaging to estimate the sensitivity profile [13]. However, neither of these techniques work well for the flexible array coils applied in cardiac imaging. In the third technique, the coil sensitivity profile is derived directly from the image series itself, using the baseline signal from the perfusion sequence images prior to the arrival of contrast agent [14]. In the fourth method, coil sensitivity profile is estimated from an additional image, typically PD-weighted, acquired prior to the actual image series [15,16]. The SSFP-SCIC and PD-SCIC techniques applied in the current study are in keeping with these latter two methods respectively. Whilst both techniques have potential for amplification of noise in regions with low coil sensitivity, we have previously demonstrated that SCIC with intensity surface fitting, as performed here, attenuates amplification of noise [7]. Furthermore we excluded regions with very low signal (e.g. lungs), where there would be potential for amplification of noise, from the intensity fit.

In two studies involving perfusion CMR in healthy volunteers, Hsu et al. demonstrated a significant reduction in the variation of semi-quantitative myocardial signal intensity parameters when a PD-weighted reference image was used to calculate surface coil related signal variation [15,16]. This is in keeping with the findings displayed in Figure 2, where time-signal intensity curves from a normal subject are considerably more similar in all segments after applying surface coil intensity correction (Panels D and G vs. A). Subsequently Kremers et al. [8] showed that segmental MBF heterogeneity in healthy volunteers fell from 29% when perfusion analysis was performed without SCIC, to 20% after SCIC was performed using a PD-weighted image acquired at the beginning of the perfusion sequence; results very similar (36% and 21% respectively) to those in the current study. Indeed, the magnitude of MBF heterogeneity in healthy volunteers in the current study is in keeping with an estimation of natural MBF heterogeneity (16%), based on animal data, that Kremers et al. performed. In the current study PD-SCIC led to a more homogenous correction than SSFP-based SCIC, most likely because the original (i.e. before SCIC was applied) PD-weighted image had higher signal intensity than the baseline saturation prepared SSFP image which aided the estimate of the intensity bias field.

The current study demonstrates, for the first time, how surface coil-related field inhomogeneity can compromise the ability of MBF quantification to detect CAD. In a third of patients with CAD, perfusion analysis without SCIC resulted in an erroneous regional pattern of MBF, with MBF being measured higher in the stenosed coronary territory compared to the remote territory. This particularly affected the detection of LAD ischaemia, which most likely reflects the previously described surface coil-related regional variation in signal intensity (higher in the anteroseptum and anterior wall and lower in the inferior and inferolateral walls). Whilst PD-SCIC led to a more homogenous correction of myocardial signal intensity and lower MBF heterogeneity in healthy volunteers, this did not translate into improved differentiation of ischaemic from remote coronary territories. A possible reason for this is that the higher heterogeneity associated with SSFP-SCIC was distributed randomly across the myocardium, thus not impacting on regional variations in MBF estimates.

The process of quantifying MBF from perfusion CMR images can be elaborate and involves a number of methodological steps, including myocardial border delineation, image registration, extraction of left ventricular blood pool (AIF) and myocardial time-signal intensity curves, conversion of signal intensity to contrast concentration, first-pass contrast timing point detection and deconvolution modelling. Whilst many perfusion CMR quantification studies have used spoiled gradient echo perfusion sequences which may be less susceptible to surface coil field inhomogeneity than SSFP sequences, SCIC is rarely mentioned in the methodology of studies involving CMR perfusion quantification [1,17-19]. This study demonstrates that SCIC is another important step that requires incorporation into the quantification algorithm.

### Limitations

The number of patients included was relatively small, however the sample size was considerably larger than any previous study evaluating SCIC in cardiac perfusion analysis. Patients with CAD were selected on the basis of having ≥70% coronary luminal narrowing as determined visually. The limitations of visual assessment of angiography are well recognized, however any inaccuracy in stenosis assessment would have impacted on MBF quantified using each SCIC technique equally. In the absence of an external MBF imaging reference (e.g. positron emission tomography), which would have allowed the true extent of ischaemic myocardium to be identified, standardised coronary territories were used for objectivity and consistency. However, this inevitably minimised the apparent difference in MBF between remote and ischaemic territories, because stenosed coronary arteries almost never lead to ischaemia of their entire standard territories and nothing more. For example, an RCA stenosis would rarely lead to ischaemia of the entire inferior and inferoseptal segments and nothing more. In reality an RCA stenosis may affect part of these segments, or extend beyond these segments. Nevertheless, this would have affected MBF quantified using each SCIC technique equally.

## Conclusions

This study demonstrates that surface coil-related field inhomogeneity can confound pixel-wise MBF quantification. It is therefore important that surface coil intensity correction is incorporated into perfusion quantification algorithms. Whilst a proton density-based surface coil intensity correction led to a more homogenous correction than a saturation recovery SSFP-based technique, this did not result in an appreciable difference in the differentiation of ischaemic from remote coronary territories and thus either method could be applied.

## Abbreviations

AIF: Arterial input function
CAD: Coronary artery disease
CMR: Cardiovascular magnetic resonance
MBF: Myocardial blood flow
No-SCIC: Perfusion analysis performed without surface coil intensity correction
PD: Proton density
PD-SCIC: Proton density-based surface coil intensity correction
ROI: Region of interest
SCIC: Surface coil intensity correction
SSFP: Steady-state free precession
SSFP-SCIC: Saturation recovery SSFP-based surface coil intensity correction
